# Reports on the Hospitals of the United Kingdom

**Published:** 1909-10-23

**Authors:** Henry Burdett


### REPORTS
ON THE
### Hospitals of the United Kingdom.

By SIR HENRY BURDETT, K.C.B., K.C.V.O.

### IV.—SOME WELSH HOSPITALS.

### CARDIFF INFIRMARY.

This hospital was founded in 1837 and rebuilt in 1882. It occupies a prominent site, and externally is one of the most attractive features of the town. It contains 190 beds, and is subject to great pressure at times, and much active and good work is carried on here. The new out-patient department just completed is an ambitious attempt to bring this portion of the work up to date. There is a very elaborate eye department, which is a wonderful development upon everything heretofore attempted in Wales.

Taken as a whole the hospital represents an average efficiency for a hospital of its date. Parts of it are very good, parts of it are defective, and some of the wards are bad in the sense that they have no lavatories and conveniences attached to them. The committee and medical staff are fully alive to these defects, and steps are being taken to remedy them, as the Chairman of the Medical Board advised us. Judged by the highest standard of administration the various portions of the hospital lack smartness; a hospital of this size and importance must be treated critically; so, although we have found many good features—for instance, the fine children's ward, which is large and well-kept and wisely managed, there were many things in various places which seemed to us to point to an absence of that close attention to detail which in the whole makes for hygienic efficiency. For instance, the theatre, a comparatively recent addition, has been added on to the old theatre, and is in direct communication with it. The new theatre is, in our judgment too large, and it cannot be said to be aseptic, for it has an open barrel drain at one side, under the stair leading to the spectators' platform. The accommodation for the nursing staff represents the good, the bad and the medium of efficiency. For instance, the sisters have probably the finest bed-sitting-rooms of almost any British hospital. Some of these rooms are bowers of attractive design. The colours and decorations are excellently chosen, and most attractive. The nurses, however, are housed in cubicles some of which are open at the top, are not provided with an entire window, and are altogether unworthy of Cardiff. Another type of the so-called cubicle is an improvement, for these are not open at the top, and are a standing proof that the worst type of cubicle in this hospital cannot be commended on hygienic, economical or moral grounds. The best type of sleeping accommodation for nurses is good, and we hope that the whole of the nurses' quarters may before long be averaged up to this standard.

The existing verandahs to the wards are too narrow, and should be broadened and extended the whole length of each ward. They should also have glass roofs, so that the patients may be kept out day and night in suitable cases. The verandahs in any case are useful, but this fact illustrates the wisdom of widening and extending them throughout the hospital. Unfortunately the medical officer, Mr. Wallace, was very ill, and we were unable to see him.

The resident medical staff is unusually large, consisting of a house physician and four house surgeons, but the work gains much, and the efficiency which it renders possible wholly justifies the expense. The Matron, Miss E. A. Mont-Wilson, has had a distinguished career, having done good service in South Africa, and been Matron of this institution for many years. We are greatly indebted to her and to Mr. J. Tatham Thompson, the deputy chairman of the Board, for their great kindness and courtesy in explaining the work and the working of the hospital during our visit.

### NEWPORT AND MONMOUTHSIRE HOSPITAL.

Built in 1901 from plans by Mr. R. J. Lovell, Mr. Pennington acting as adjudicator. It is just an H plan with the administration block in the centre. It does not exhibit expert knowledge, nor appreciation of hospital requirements in the economic and hygienic sense. Economically space has been wasted, whereby the cost of upkeep has been increased. This defect has added nothing to the completeness and smart appearance of the hospital, but quite the contrary. The ward units are not so well arranged or thought out as one has a right to expect in a hospital so recently built. This criticism applies to the operation theatre, which has neither anæsthetic, recovery, nor sterilising rooms, nor the facilities of an up to date hospital. The same remarks apply to the kitchen unit, and indeed to the whole plan, from the administrator's point of view.

We went through the hospital, which stands upon an extended site with ample grounds, on a Saturday in the late afternoon. It contains all the essentials of a hospital, but it is not one which gives a good and bright impression to the visitor. Allowance must be made for the hour of the visit, and the circumstances resulting, but when this is granted it is but just to say that the elevation gave us hopes of a modern up to date hospital which were not realised by an inspection of the hospital itself. Miss Evans, the matron, was interested in her work, and most courteous and kind, and we greatly appreciated her courtesy.

### ROYAL HAMADRYAD SEAMEN'S HOSPITAL, CARDIFF.

This hospital was founded in 1866. It was transferred from the ship to a specially erected building on shore about four years ago. In March 1905 the King commanded that the institution should be styled "Royal." It contains 54 beds, and relieves some 600 in-patients and some 8,000 out-patients, largely foreign seamen. The new hospital is well planned, well found and well kept throughout. The day room on each floor facing the water is quite excellent. We made a very detailed and careful inspection, being attracted to it by the smart appearance and the excellent spirit which prevailed throughout the institution. It is certainly well managed, and displays much frugality and method in its arrangements. It has a remarkably small staff, which must be specially efficient. The Matron with the help of one Sister and five probationers provides the nursing for 54 beds. Extra help is obtained when the cases are particularly heavy. The washing is done in a small laundry by one woman with a man to help her three days a week. Again, one cook assisted by a scullery maid does all the cooking. Economy with efficiency could hardly be carried further than this, for we found everything well kept and in good order. It would be well if the same spirit which has produced such splendid results at the Royal Hamadryad Hospital could spread to every hospital throughout Wales. Several of these other hospitals contain much which is good, but undoubtedly lack the Hamadryad spirit, to their incalculable loss.

We noticed one or two small points which should have attention. The heating radiators in the wards are defective, and unless they can be made perfectly efficient the firm which supplied them should be called upon to make them good. The larder and linen store both need ventilation badly. Having regard to the exceptional excellence of this hospital we were sorry to find that it receives little or no support from the people of Cardiff, and the district. It has a claim on all landsmen, and we strongly recommend the committee at once to set to work vigorously to raise an additional £500 per annum income, which is badly needed. A very little effort, if the appeal is made wide enough, and if it includes with the aid of the press the inland towns of England and Wales, and yachtsmen, would produce this additional revenue of £500. We congratulate the committee, and especially Dr. Whelan, the Medical Superintendent, and Miss Davies, the Matron, upon the present state of this hospital, which does them the greatest credit, and should win for them not only the gratitude of seamen but that of all who take an interest in the hospitals of Cardiff and of this hospital in particular.

**The Queen's Hospital, Birmingham,** and **Wolverhampton Eye Hospital,** will be reported on next week.

